# Dose-response studies of Ropivacaine in blood flow of upper extremity after supraclavicular block: a double-blind randomized controlled study

**DOI:** 10.1186/s12871-017-0447-7

**Published:** 2017-12-02

**Authors:** Ting Li, Qiguang Ye, Daozhu Wu, Jun Li, Jingui Yu

**Affiliations:** 1 Department of Anesthesiology, Qilu Hospital of Shandong University, Jinan, China; 2 Department of Anesthesiology, The Second Affiliated Hospital and Yuying Children Hospital of Wenzhou Medical University, Wenzhou, China; 3 Ultrasonic Department, The Second Affiliated Hospital and Yuying Children Hospital of Wenzhou Medical University, Wenzhou, China

**Keywords:** Brachial plexus, nerve block, Local anesthetic, Hemodynamics, Upper extremity

## Abstract

**Background:**

The sympathetic block of upper limb leading to increased blood flow has important clinical implication in microvascular surgery. However, little is known regarding the relationship between concentration of local anesthetic and blood flow of upper limb. The aim of this dose–response study was to determine the ED_50_ and ED_95_ of ropivacaine in blood flow after supraclavicular block (SB).

**Methods:**

Patients undergoing upper limb surgery and supraclavicular block were randomly assigned to receive 30ml ropivacaine in concentrations of 0.125%(A Group), 0.2%(B Group), 0.25%(C Group), 0.375%(D Group), 0.5%(E Group), or 0.75%(F Group) (n=13 per group). All patients received supraclavicular block (SB). Time average maximum velocity (TAMAX), cross-sectional area (CSA) of brachial artery and skin temperatures (T_s_) were measured repeatedly at the same marked points, they were taken at baseline (before block, t_0_) and at 30min after SB (t_1_). Blood flow(BF) = TAMAX× CSA×60 sec.. Relative blood flow (ΔBF) = BF_t1_/ BF_t0_. Success of SB was assessed simultaneously. Supplementary anesthesia and other adverse events (AE) were recorded.

**Results:**

Significant increase in TAMAX, CSA, BF and T_s_ were seen in all concentration groups at t_1_ comparing with t_0_ (*P<*0.001). There was an upward trend of TAMAX, CSA, BF with the increasing concentration of ropivacaine except T_s_. There was no significant different of T_s_ at t_1_ among different concentration group. The dose-response formula of ropivacaine on ΔBF was Y=1+3.188/(1+10^((−2.451-X) × 1.730)) and ED_50_/ED_95_ (95%CI) were 0.35/1.94%(0.25–0.45/0.83–4.52), and R^2^ (coefficient of determination) =0.85. ED_50_/ED_95_ (95%CI) values of sensory block were 0.18/0.33% (0.15–0.21/0.27–0.51), R^2^=0.904.

**Conclusions:**

The dose-response curve between SB ropivacaine and the changes of BF was determined. The ED_50_/ED_95_ of ropivacaine of ΔBF are 0.35/1.94% (0.25–0.45/0.83–4.52). TAMAX, CSA and BF consistently increased with ropivacaine concentration. The maximal sympathetic block needs higher concentration than that complete sensation block needs which may benefit for microvascular surgery.

**Trial registration:**

Clinicaltrials.gov NCT02139982. Retrospectively registered (Date of registration: May, 2014).

## Background

Brachial plexus block (BPB) is widely used in regional anesthesia [1]. Its sympathetic block leads to vasodilatation and increased blood flow and skin temperature in the ipsilateral upper limb [2–4]. So this technique is especially useful for microvascular surgery such as replantation of digits. Currently, there are several literatures of regional hemodynamic changes of arteriovenous fistula after a BPB [5–7]. The role of increased blood flow after BPB is considered to be of great importance for microvascular surgery.

Recent publications have reported dose-finding studies of sensory block in brachial plexus and given the ED_95_ or ED_50_ dose [8, 9]. However, there is no literature about dose-finding studies of sympathetic block in brachial plexus. Little is known regarding the relationship between concentration of local anesthetic and blood flow of upper extremity. The aim of this randomized, double-blind, concentration–response study was to determine the ED_50_ and ED_95_ of ropivacaine in blood flow of upper extremity after supraclavicular block.

## Methods

This double-blind randomized controlled, dose-response study was conducted in China. Study approval was obtained from the Institutional Review Board (IRB) of the Second Affiliated Hospital and Yuying Children Hospital of Wenzhou Medical University (109 Xueyuang xi Road, Wenzhou, China, Ref 2009(002), February 2th, 2009, Prof. Qinquan Lian). This trial was registered at Clinicaltrials.gov with the identifier NCT02139982 on 28 May 2014. Informed consent was obtained from each patient. Between July 2011 and June 2012, all consecutive adults undergoing upper extremity surgery were screened for the trial. Inclusion criteria were adults (>18-year-old), American Society of Anesthesiologists (ASA) physical status I-III and elective surgery. Exclusion criteria were infection at the site of needle insertion; any coagulopathy; allergy to local anesthetics; peripheral neurological disease, or peripheral vascular disease. Patients were randomly assigned to receive 30 ml ropivacaine (Naropin, Astra-Zeneca, Sweden) in concentrations of 0.125%(A Group), 0.2%(B Group), 0.25%(C Group), 0.375%(D Group), 0.5%(E Group), or 0.75%(F Group) using computer (Microsoft excel 2010) generated random number in sealed envelopes.

Each patient lay supine in a hospital bed in the recovery room with room temperature of 24 °C, acclimatizing for 15 min. All patients were performed routine monitoring (NIAP, continuous ECG, and pulse oximetry). All nerve blocks were performed by a single dedicated anesthetist. Time average maximum velocity (TAMAX), cross-sectional area (CSA) of brachial artery and skin temperatures (T_s_) were measured repeatedly at the same marked points at baseline (before block, t_0_) and at 30 min after SB (t_1_). TAMAX is a sensitive hemodynamic index, increased with the vasodilatation and the reduction of peripheral vascular resistance. CSA is a direct index of vasodilatation. Heart rate (HR) and mean arterial pressure (MAP) were recorded simultaneously. All these measurements were performed by another investigator who does not know the concentration of ropivacaine. For patients with an ineffective block before surgery, supplementary anesthesia (conversions to general anesthesia, supplementary brachial plexus block or branch nerve block with 10–20 ml of ropivacaine 0.5%) was administered according to the site of surgery and anesthetist’s experience. If the patient experienced any pain during his surgery, supplementary analgesia (fentanyl 1 to 2 μcg/kg), sedation (midazolam 10 to 30 μcg/kg), or general anesthesia was administered as required. Supplementary anesthesia and other adverse events (AE) were recorded.

### Supraclavicular block

A single anesthetist with 7 years experience of ultrasound-guided nerve blocks performed all brachial plexus blocks. The brachial plexus was visualized with a high-frequency linear ultrasound transducer (HFL 38X/13–6 MHz, S-nerve TM Ultrasound System, SonoSite Inc., USA) in the supraclavicular fossa. After disinfection and infiltrating with 1% lidocaine, by in-line technique, nerve stimulation needle (Stimuplex® D, 22G, 50 mm; B.Braun Melsungen AG, Germany) was inserted and advanced among the divisions of brachial plexus with electric impulses (2 Hz, 0.5 mA, 0.1 ms) of nerve stimulator (Stimuplex® HNS 11 Peripheral Nerve Stimulator, B.Braun Melsungen AG, Germany). If visible contraction of the innervated muscle was elicited, the needle was withdrawn slowly until the corresponding muscle contraction disappeared to avoid intrafascicular puncture [10]. The local anesthetic was injected at three locations: adjacent to the superficial divisions of the plexus, adjacent to the middle divisions and adjacent to inferior divisions. The spread of local anesthetic was observed to ensure the quality of the performance of BPB. The proportion of the volume injected in each area was at the discretion of the expert operator according to the spread of local anesthetic. This operator remained blind to the concentration of ropivacaine.

### Block assessment

Another investigator, who was not present during the conduct of the SB and was completely blinded to the concentration of ropivacaine used, assessed each blockade. The subjects did not know the concentration of local anesthetic used either. The efficacy of the block was assessed by pinprick sensation (22 G needle) and compared with the opposite forearm/hand and recorded as sensation, hypoesthesia or no sensation at 30 min after the SB. Success of SB was defined as the absence of sensation in all innervation areas of four nerves (musculocutaneous, ulnar, radial, and median nerves) 30 min after the SB and no pain during the surgery.

### Measurement of hemodynamic parameters

**Fig. 1:**
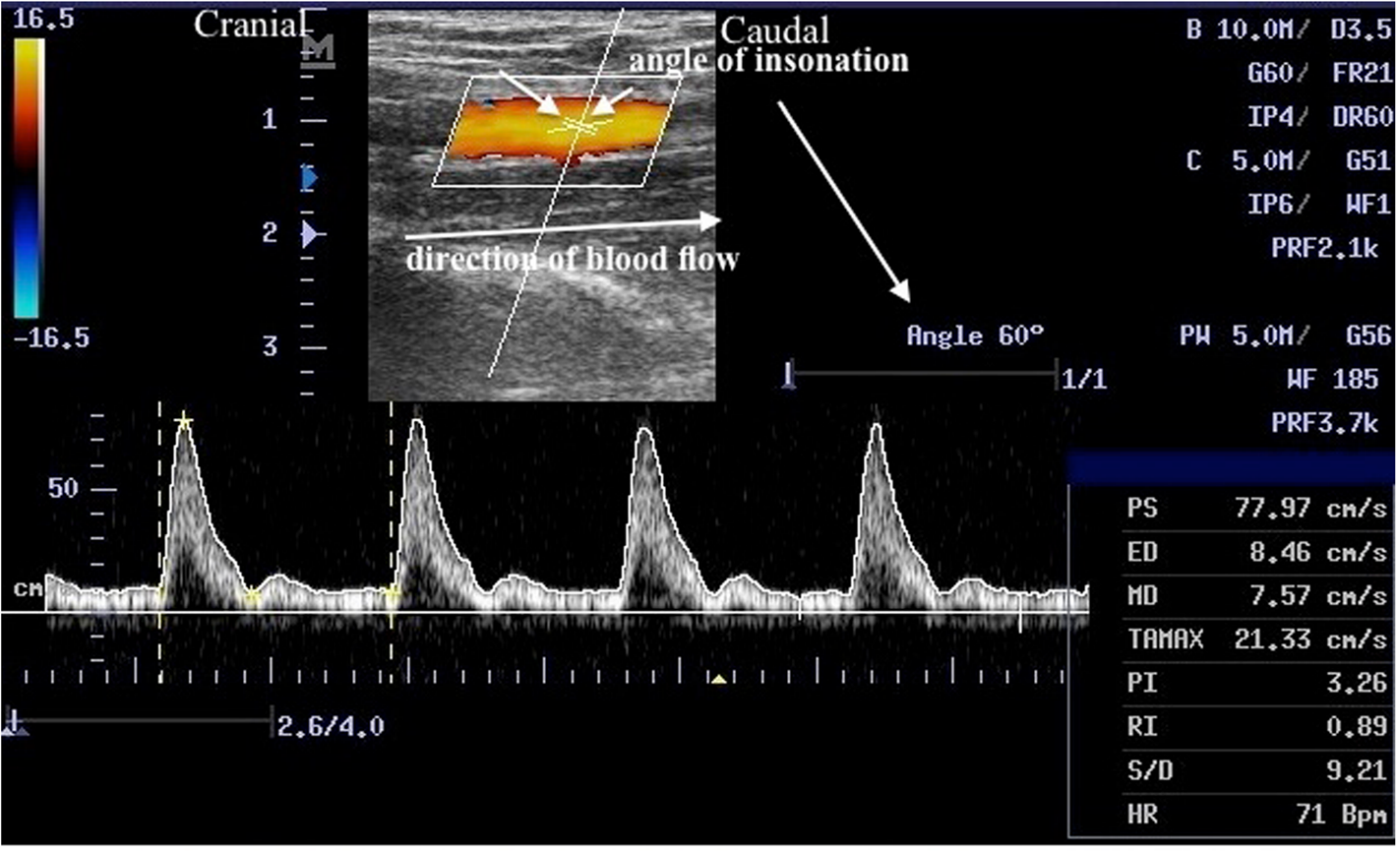
PWD Ultrasound of the brachial artery after brachial plexus block. PS: peak systolic velocity (cm/s.). ED: end-diastolic velocity (cm/s.). TAMAX: time average maximum velocity (cm/s.)

Patient’s ipsilateral forearm was in supination. The brachial artery was located in 2 cm proximal to the antecubital fossa. Specific points were located with skin marker to provide consistency with all measurements taken. Hemodynamic Parameters were measured by Pulsed-wave Doppler (PWD) ultrasound (DC-6, Mindray Medical International Limited, China) with a 7 L4 linear array transducer (frequency 10 MHz). The probe was parallel the long axis of the arm without undue pressure on the artery during the PWD measurements. The volume gate was positioned in the center of the arterial lumen, and the size of the gate was 1/3 lm of the artery. The angle of insonation was adjusted and maintained at 50–60 degrees. Once a desired PWD spectral waveform was achieved, the arterial TAMAX was recorded. (Fig. 1). CSA of the artery was assessed with B-mode imaging. Probe should be as perpendicular as possible to the long axis of the artery to obtain as round an arterial section as possible. The image at end diastole was chosen with the cine loop. The changes in arterial cross-sectional area during the cardiac cycle was not measured in this study. BF was calculated by formula: BF = TAMAX× CSA × 60s. Relative blood flow (ΔBF) = BF_t1_/ BF_t0_.

### Measurement of temperature

Skin temperature (T_s_) was measured at the thenar by the rapid precise contact thermometer (DAE-905 T, ShengGao Inc., China). Specific points were located with skin marker to provide consistency of measurement.

### Statistical analysis

GraphPad Prism for Windows 5.0 (GraphPad Software, Inc., San Diego, USA) was used for nonlinear regression to build dose-response of ΔBF. The equation of nonlinear regression is:

$$ Y\kern0.5em = Bottom+\kern0.5em \frac{\left( Top- Bottom\right)}{1+{10}^{\kern0.5em \left(\log \kern0.5em {EC}_F-X\right)\kern0.5em \times \kern0.5em HillSlope}} $$

Where *X* is the logarithm of concentration of ropivacaine, *Y* is the relative blow flow ΔBF, *F* is a constant value between 0 and 100, *Top* is the Y value at the top plateau, *Bottom* is the Y value at the bottom plateau, is the X value when the response is halfway between *Bottom* and *Top* and *HillSlope* is the steepness of the curve.

Personal information, surgical details, MAP, HR, hemodynamics and T_s_ were collected and the data were presented as mean (SD) or numbers as appropriate. Paired-Samples T test was applied to compare hemodynamic parameters and skin temperature before and after brachial plexus block, Kruskal-Wallis H non-parametric test was applied to compare these variables among different concentration groups. Analysis of variance trend test and Jonckheere’s trend test were performed to detect the trend in changes of hemodynamic parameters and skin temperature as the concentration increased. Probit regression was used to calculate the ED_50_ and ED_95_ of ropivacaine on sensory block. For all tests, significance was defined as a *p* value <0.05. Statistical analysis was conducted using SPSS for Windows 16.0 (SPSS Inc., Chicago, USA).

## Results

**Fig. 2:**
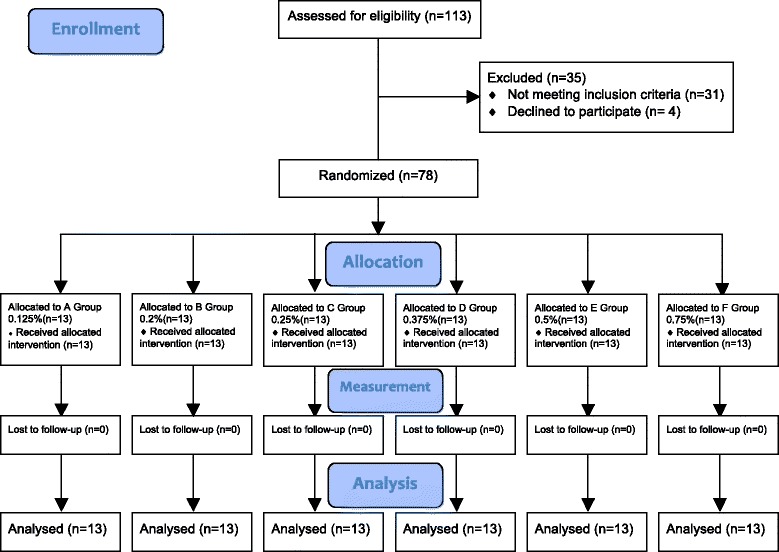
The CONSORT flow diagram for randomized controlled trials

The subjects approached are summarized using a CONSORT flow diagram (Fig. 2). All patients were undergoing elective surgery (24 removal of metalwork from ulnar/radial, 18 fixation of fractured ulnar/radial, 12 fixation of fractured metacarpal, 11 fractured phalanx, 6 amputation of finger and 7 surgical removal of soft tissue mass). There was no significant change detected in the heart rate and blood pressure after the SB across the six groups (Table 1).

**Table 1:** Patients characteristics and changes of MAP and HR in different concentration groups at baseline (t_0_) and 30 min after brachial plexus block (t_1_)

		Groups(*n* = 13)					
		A	B	C	D	E	F
Male/female		6/7	8/5	7/6	8/5	9/4	9/4
Age (yr)		37(14)	37(13)	43(16)	36(15)	39(16)	44(17)
Height (cm)		163(7)	166(6)	165(7)	164(5)	167(7)	169(8)
Weight (kg)		58(9)	60(9)	62(8)	61(10)	62(7)	62(8)
MAP	t_0_	86(13)	87(5)	85(12)	85(14)	86(10)	85(13)
	t_1_	86(13)	87(5)	86(12)	84(12)	86(9)	87(14)
HR	t_0_	75(5)	67(8)	72(8)	69(8)	67(7)	68(8)
	t_1_	75(5)	66(8)	71(8)	70(7)	65(8)	66(8)

Values are number or mean (SD)

*MAP* Mean Arterial Pressure, *HR* Heart Rate

### Measurements of hemodynamic parameters and skin temperature

**Fig. 3:**
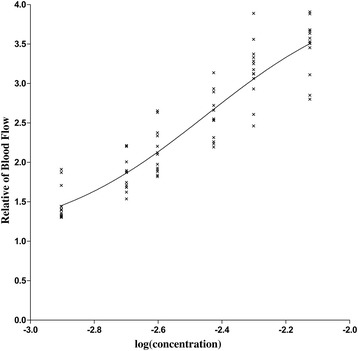
The dose-response curve of value of ΔBF (BFt1/ BFt0) against the log (concentration) of ropivacine and scattergram for supraclavicular block

Changes in TAMAX, CSA and BF of the brachial artery and T_s_ were summarized in Table 2. Significant increases in TAMAX, CSA, BF and T_s_ were seen in all concentration groups at t_1_ comparing with t_0_ (*P <* 0.001). There were not significant difference in TAMAX, CSA, BF and T_s_ at t_0_ among different concentration groups (*P >* 0.05). TAMAX, CSA and BF consistently increased with concentration (*P <* 0.05) while Ts increased monotonically from the 0.125% group to the 0.5% group (*P <* 0.05) then remained as the concentration reached 0.75%. The dose-response curve and data of ropivacaine on ΔBF was shown in Fig. 3. The dose-response formula was

**Table 2:** Hemodynamic parameters of brachial artery and temperature of skin in different concentration groups at baseline (t_0_) and 30 min after brachial plexus block (t_1_)

		Groups(n = 13)					
		A	B	C	D	E	F
TAMAX(cm/s)	t_0_	23.1(5.6)	20.9(5.9)	19.6(5.6)	19.1(6.0)	18.7(8.4)	19.1(7.0)
	t_1_	29.7(7.5)^*^	33.6(8.1)^*^	35.5(8.1)^*^	39.1(10.6)^*^	44.8(18.8)^*^	49.3(19.9)^*^
CSA(cm^2^)	t_0_	0.126(0.025)	0.140(0.056)	0.133(0.031)	0.131(0.035)	0.114(0.028)	0.114(0.038)
	t_1_	0.145(0.032)^*^	0.159(0.062)^*^	0.155(0.038)^*^	0.162(0.042)^*^	0.149(0.038)^*^	0.155(0.055)^*^
BF(ml/s)	t_0_	178.2(62.5)	175.9(93.6)	158.2(65.0)	148.6(59.8)	127.6(61.7)	135.5(71.3)
	t_1_	259.3(87.7)^*^	320.3(152.0)^*^	330.4(118.4)^*^	378.7(143.9)^*^	399.7(190.9)^*^	465.2(240.6)^*^
Ts(°C)	t_0_	31.0(1.4)	29.6(1.1)	30.2(1.4)	29.5(1.3)	29.5(1.1)	29.8(0.8)
	t_1_	32.2(1.2)^*^	31.5(0.9)^*^	32.5(1.2)^*^	31.8(1.3)^*^	31.9(1.2)^*^	32.2(1.2)^*^

Mean and SD values rounded to 2 decimal places

*P* values are results of 2-tailed, paired student *t* tests. ^*^ *P* < 0.01 compared with t_0_

*TAMAX* time average maximum velocity (cm/s), *CSA* cross section area, *BF* blood flow, *Ts* skin temperature

$$ Y\kern0.5em =\kern0.5em 1+\frac{3.188}{1+10\left(-2.451-x\right)\kern0.5em \times \kern0.5em 1.730} $$

ED_50_/ED_95_ (95% CI) values of ΔBF were 0.35/1.94%(0.25–0.45/0.83–4.52), and R^2^(coefficient of determination) =0.85.

### Assessment of sensory block

**Fig. 4:**
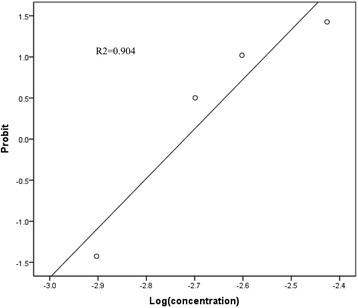
Linear regression plot of the probit value against the log (concentration) of ropivacine for supraclavicular block

The successful rates of sensory block in different concentration groups were: 1/13(A group), 9/13(B group), 11/13(C group), 12/13(D group), 13/13(E group), 13/13(F group) respectively. Fig. 4 showed the predicted concentration–response curves of ropivacaine on sensory block of SB. ED_50_/ED_95_ (95% CI) values were 0.18/0.33% (0.15–0.21/0.27–0.51) and R^2^ = 0.904.

### Adverse event

The rate of supplementary anesthesia in different concentration groups were: 11/13(A group), 2/13(B group), 1/13(C group), 0/13(D group), 0/13(E group), 0/13(F group). Total 5 patients were administered supplementary analgesia and sedation in A-D group. Two (2/13) and (1/13) of Horner syndrome occurred in F group and D group respectively. One (1/13) patient presented hoarseness in F group. All these AE was recovered. No dyspnea or symptoms of local anesthetic toxicity was reported in all group.

## Discussion

In this study, changes in TAMAX, CSA, BF were seen in all concentration groups with an upward trend as the concentration increased. Compared to baseline, BF increased from about 1.5-fold to 3.5-fold with the concentration of ropivacine increased. To minimize the bias of individual different, we used ΔBF to build the dose-response curve. The estimated ED_50_/ED_95_ of ΔBF were 0.35/1.94%. It means that using these concentrations will get 50/95% the extreme blood flow. Simultaneously the sensory block was assessed to build dose-response curves by probit analysis. ED_50_/ED_95_ values of sensory block were 0.18/0.33% which were similar to Fredrickson’s study [11]. It means these concentrations make 50/90% patients lose sensation. Obviously, the ED_50_/ED_95_ of ΔBF are higher than ED_50_/ED_95_ of sensory block respectively. The ED_95_ of sensory block is equivalent to the ED_50_ of ΔBF. This result indicates that sensory block is achieved by lower concentration of ropivacaine than complete sympathetic block. High concentration of ropivacaine is required to maximize sympathetic block. We may administer higher concentration of local anesthetic than normal postoperative analgesia concentration (0.2%) to get more vasodilatation and reduction of peripheral vascular resistance which may benefit for microvascular surgery. However, the extrapolating ED_95_ of ΔBF is higher than the maximum trial concentration (ropivacaine 0.75%), its value may not be reliable. Additionally, both dose-response curves have high R^2^ value, means they are with well fitted.

There is not a consistent upward trend of T_s_ following the increasing concentration. So we didn’t build the dose-response curve of T_s_. Though the rising T_s_ is a result of the increase of skin blood flow, T_s_ is affected by many factors. Thus we believe that the blood flow is a more sensitive index for intensity of sympathetic block in upper limb than T_s_. And also T_s_ rises significantly with very low successful rate of sensory block in the lowest concentration (0.125%). It agrees with Hermanns’s study that T_s_ is of limited clinical value to predict the success of sensor block during interscalene blockade [12].

Bigeleisen’s [10] study found if stimulation threshold was greater than 0.5 mA, the possibility of intrafascicular puncture was very little. In this study, we use ultrasound combined with nerve stimulate technique to block brachial plexus, and assure no visible contraction of the innervated muscle was elicited at 0.5 mA electric impulses to avoid intrafascicular puncture and to make the effect of block steady. we have not find an abnormal rapid blockade in all patients in fact.

A tightly controlled laboratory study in animals showed that the intensity of nerve blockade was primarily determined by local anesthetic concentration [13]. So, this study was designed to build dose-response curve with the fixed volume of local anesthetic. Kant’s [8] study estimated that the ED_95_ dose of 0.5% Bupivacaine for Ultrasound-guided Supraclavicular Block was 27 ml. Fredrickson’s [11] study reported the ED_95_ dose of 0.5% ropivacaine for interscalene block was 20.5 ml. However, the ED_95_ of volume may be different according to different concentrations and different approaches to brachial plexus block. Michał’s [11] study recommended ultrasound combined nerve stimulator guided blocks (the dual guidance technique) to reduce the risk of complications for shoulder surgery with 20 mL of 0.5% ropivacaine. Vandepitte [14]reported that a minimum of 7 ml of ropivacaine 0.75% through the catheter is required for success rate and timely onset of surgical anesthesia with interscalene brachial plexus blockade. No study has reported the exact ED_95_ of these concentration groups in Supraclavicular Block. In our study, we used 30 ml local anesthetic for Supraclavicular Block in all concentration groups. Lower volume would probably be enough to produce a surgical block in groups 0.375%–0.75% but in groups with 0.125% ­ 0.25% ropivacaine BPB, would probably require additional sedation, analgesia or conversion to general anesthesia. The assumed primary benefit of BPB for the patient was not only to improve the blood flow in the brachial artery and but also secondly provide a perioperative analgesia rather than perform the surgical block. Additionally, some centers like our hospital prefer combination of sedation or general with regional anesthesia over general or regional anesthesia only. [15].

Currently; PWD ultrasound is a common method to measure the Hemodynamic changes in the upper extremity [16]. Our study investigated the sympathetic effects of brachial plexus block in terms of Hemodynamics by PWD ultrasound and T_s_. The authors took many steps to minimize measurement errors, including controlling the ambient temperature and humidity; having all measurements performed by a single investigator; using a standardized measurement of PWD ultrasound and marking the position of measurement.

However, this study suffers from some limitations. Firstly, we cannot totally avoid the intrafascicular injection that usually causes abnormal and complete block. Secondly, we are not sure that 30 min is enough for each concentration group to achieve maximal effect of block. Future clinical studies should focus on the optimal concentration of ropivacaine for continuous BPB with the most benefit for microvascular reconstruction surgery and minimal risk of toxicity.

## Conclusion

In summary, the dose-response curve between SB ropivacaine and the changes of BF was determined. The ED_50_/ED_95_ of ropivacaine of ΔBF are 0.35/1.94%(0.25–0.45/0.83–4.52). TAMAX, CSA and BF consistently increased with ropivacaine concentration. The maximal sympathetic block needs higher concentration than that complete sensation block needs which may benefit for microvascular surgery.

## Abbreviations

AE: Adverse events
ASA: American Society of Anesthesiologists physical status
BF: Blood flow
BPB: Brachial plexus block
CSA: cross-sectional area
ED: End-diastolic velocity
HR: Heart rate
IRB: Institutional Review Board
MAP: Mean arterial pressure
PS: Peak systolic velocity
PWD: Pulsed-wave Doppler
SB: Supraclavicular block
TAMAX: Time average maximum velocity
Ts: Skin temperatures
ΔBF: Relative blood flow
